# A Low-Cost NDIR-Based N_2_O Gas Detection Device for Agricultural Soils: Assembly, Calibration Model Validation, and Laboratory Testing

**DOI:** 10.3390/s21041189

**Published:** 2021-02-08

**Authors:** K.M.T.S. Bandara, Kazuhito Sakai, Tamotsu Nakandakari, Kozue Yuge

**Affiliations:** 1 United Graduate School of Agricultural Sciences, Kagoshima University, 1-21-24 Korimoto, Kagoshima-shi, Kagoshima 890-0065, Japan; thusihta@gmail.com (K.M.T.S.B.); zhunai@agr.u-ryukyu.ac.jp (T.N.); yuge@cc.saga-u.ac.jp (K.Y.); 2 Department of Agricultural Engineering, Faculty of Agriculture, University of Ruhuna, Kamburupitiya 81100, Sri Lanka; 3 Faculty of Agriculture, University of the Ryukyus, 1 Senbaru, Nishihara-cho, Okinawa 903-0213, Japan; 4 Faculty of Agriculture, Saga University, 1 Honjo-machi, Saga 840-8502, Japan

**Keywords:** NDIR, N_2_O gas sensor, low-cost gas monitoring, gas sensor calibration, soil Nitrous Oxide emission, silicone diffusion cell

## Abstract

This research presents a low-cost, easy-to-assemble nondispersive infrared (NDIR) device for monitoring N_2_O gas concentration in agricultural soils during field and laboratory experiments. The study aimed to develop a cost-effective instrument with a simple optic structure suitable for detecting a wide range of soil N_2_O gas concentrations with a submerged silicone diffusion cell. A commercially available, 59 cm path-length gas cell, microelectromechanical systems (MEMS)-based infrared emitter, pyroelectric detector, two anti-reflective (AR) coated optical windows, and one convex lens were assembled into a simple instrument with secure preciseness and responsivity. Control of the IR emitter and data recording processes was achieved through a microcontroller unit (MCU). Tests on humidity tolerance and the saturation rate of the diffusion cell were carried out to test the instrument function with the soil atmosphere. The developed calibration model was validated by repeatability tests and accuracy tests. The soil N_2_O gas concentration was monitored at the laboratory level by a specific experimental setup. The coefficient of determination (R^2^) of the repeatability tests was more than 0.9995 with a 1–2000 ppm measurability range and no impact of air humidity on the device output. The new device achieved continuous measuring of soil N_2_O gas through a submerged diffusion cell.

## 1. Introduction

Population growth demands a higher rate of crop production, for which the crops’ nutrient requirements are largely accomplished through the use of synthetic fertilizers [1]. Nitrogen (N) fertilizer is an essential basic component of crop nutrient inputs [2]. Nitrogen-based nutrients are mainly provided by synthetic fertilizer and subsequently, from the recycling of crop residues, animal waste and biological N fixation by legumes as organic manure [3,4]. During the past 159 years, the atmospheric nitrous oxide (N_2_O) concentration has increased from 270 to 323 ppbv due to synthetic fertilizer applications and other anthropogenic activities [5]. Nitrous oxide contributes significantly to the depletion of stratospheric ozone [6]. Due to its inherited characteristics, such as a high radiative capacity and long atmospheric lifetime, N_2_O contributes to atmospheric warming 298 times more powerfully than carbon dioxide [7]. Therefore, the reduction of N_2_O emission is highly significant and could be achieved by assessing the excessive fertilizing at agricultural fields.

In the instrumentation sector, various types of N_2_O gas-metering methodologies, including gas chromatography, chamber methods, infrared-based optical methods and laser absorption spectroscopy, have been introduced in diverse applications [8,9,10,11]. Specifically, a portable instrument needs to be located at agricultural fields to determine the nitrous oxide flux in long-term data acquisition [9,10]. Concerning applications in field conditions, the said metering methodologies have some negative aspects. Chromatography requires sample collection (less portability), and the FTIR optical method is expensive and associated with difficulties around maintenance. Laser absorption spectrometers are expensive, and a cryogenic cooling system is required [11]. In some laboratory experiments, soil N_2_O gas concentration monitoring under various fertilizer concentrations has encountered difficulties at a higher range of gas levels since most high precision gas analyzers can measure gases up to 500 ppm. Designing and developing a low-cost portable device for monitoring onsite N_2_O gas concentrations at agricultural fields, and conducting laboratory tests in a wide range of soil gas concentrations, will be indispensable.

The global community has an agenda to achieve the sustainable development goals (SDGs) by 2030; climate action is aiming for a decarbonized society under the Paris agreement [12]. Bioenergy will be a key factor in achieving a decarbonized society. Bioenergy is considered “carbon neutral”. However, greenhouse gases (GHGs), especially N_2_O emissions generated during farming, are not discussed in many cases [13]. Generally, the estimation of N_2_O emission from farmlands is calculated by multiplying the amount of applied N with the emission factors defined in the guidelines of the Intergovernmental Panel on Climate Change (IPCC) [14]. However, values of the emission factors vary by region and are related to specific features in agricultural activities. Accurate estimation of N_2_O emissions in agricultural activities is vital. Therefore, research into emission factors should be conducted in many countries, and especially in developing countries that lack resources for gas measurements. However, there is a lack of sufficient related research due to the difficulties with measuring N_2_O concentration under field conditions. Measuring low concentrations of the target gas in the atmospheric region adjacent to the soil is a costly operation due to the initial and maintenance costs of high precision instruments.

NDIR technology has widely adopted for gas measurements, mostly for CO_2_ and CH_4_ detection. The comparison in performance of low-cost and commercially available NDIR CO_2_ sensors have been revealed in some research, and possible modifications with accuracy improvement are also assessed [15,16,17,18]. In the structural development, NDIR gas sensors have been modified to a broad range of specific gas monitoring applications in agricultural activities [19,20], occupational settings [21,22] and the transportation sector [23]. The basic structure of the NDIR setup encompasses a target gas containing an optical tube, an infrared light source, and a specific wavelength filter with an infrared detector [24,25,26]. As the basic structure can be customized into a simplified version, designing a low-cost portable gas measuring device is possible [27,28].

The use of silicone as a sampler to absorb gases from soil and water has been recorded in several research projects [29,30,31]. Low-cost, low-precision devices can be used for N_2_O measurements, together with a soil submerged gas-permeable membrane, since the concentration of the target gas is higher in the soil gas region than in the atmosphere. In this research, we present a prototype of a low-cost, easy-to-assemble, and portable NDIR-based device for measuring N_2_O gas through a submerged silicone diffusion cell for agricultural and laboratory soil tests.

## 2. Design of the Spectrometer

**Figure 1:**
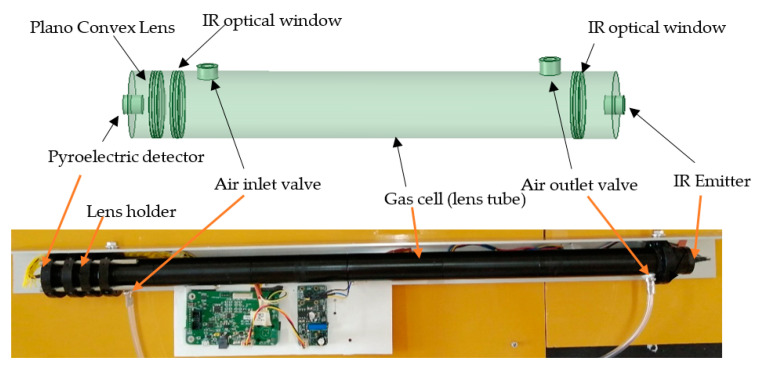
Schematic diagram of the developed gas cell with optical components and the assembled gas monitoring device.

**Figure 2:**
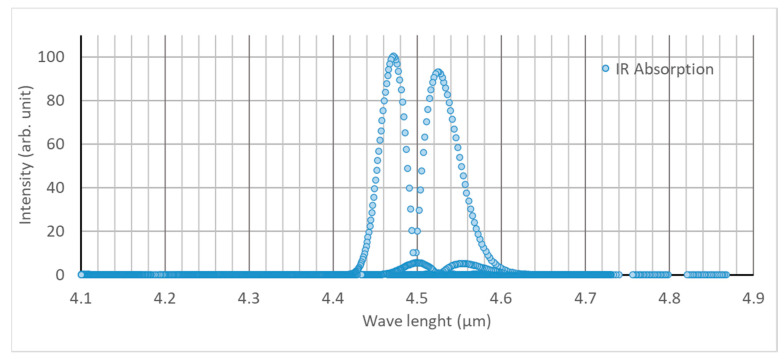
Graph of the IR absorption bands for N_2_O gas based on the data from HITRAN [32].

The design concept of the optical system included a separate gas cell, of which two edges are covered by anti-reflective (AR) coated optical windows (Thorlabs: Ø 25.4 mm, 5 mm thick, CaF_2_) (Figure 1). From the outward side of the windows, the detector and light source were connected to the gas cell. This mechanism avoids direct contact of the gas with the detector and light source, and it reduces unnecessary variations of detector output due to deposition of water vapor and other foreign particles during long term monitoring of soil gas concentration. A convex lens (Thorlabs: Ø 25.4 mm, F = 40 mm, CaF_2_, E-coated) was placed between the detector and the window to focus the light beam on a sensible point of the detector. A 60 cm long aluminum gas cell was assembled by interconnecting 25.4 mm diameter lens tubes (Thorlabs); two valves were placed at each edge for air circulation. To enhance the selectivity of the device, a bandpass filter with 4.525 µm of center wavelength (CWL) and 80 nm of half-power bandwidth (HPB) was selected since the highest levels of IR absorbance by N_2_O gas is shown at 4.47 micron and 4.52 micron wavelengths (Figure 2). A dual-channel pyroelectric detector (Pyreos, PY-ITV-DUAL-TO39) covered with a bandpass filter (4.525 µm CWL, 80 nm HPB) was applied. The MEMS-based infrared radiation source on a mounted TO39 cap with a cone reflector (Micro-Hybrid JSIR 350-4) was the light source. MCU (Pyreos PCB—C8051f350) was used with an IR emitter driver PCB (Pyreos) for the detector signal processing and IR emitter output controlling units, respectively. Recording of the rms values of the detector output and data input to control the IR emitter driver was setup using a PC-installed Pyreos graphical user interface (GUI). Two types of experiments were run to test the performance of the new device. The first experiment involved basic tests on the calibration model validation, accuracy, and repeatability of the device, as discussed in Section 3, and the other was a test of the practical usability, as discussed in Section 4.

## 3. Device Functionality Assessment

### 3.1. Test for Selecting the Operating Frequency of the IR Emitter

Since the deviations occur in the detector output with the incident light from the IR emitter that runs at varied operating frequencies, a test was conducted to determine the best operating frequency. The test was performed by recording the respective detector output for added five concentration levels of pure N_2_O gas (ranging from 0–96.1 ppm) into the N_2_ gas-filled gas chamber (volume 940 mL) while adjusting the IR emitter frequency from 2 Hz to 10 Hz. A gas-tight type microliter syringe (Hamilton 81,030 calibrated syringe) was used to inject the N_2_O gas through the rubber septum fixed on the lid of the gas chamber. The recorded rms values of detector output at each gas concentration level were graphically extrapolated with the IR emitter frequency values.

#### Results of the Operating Frequency Selection Test for the IR Emitter

**Figure 3:**
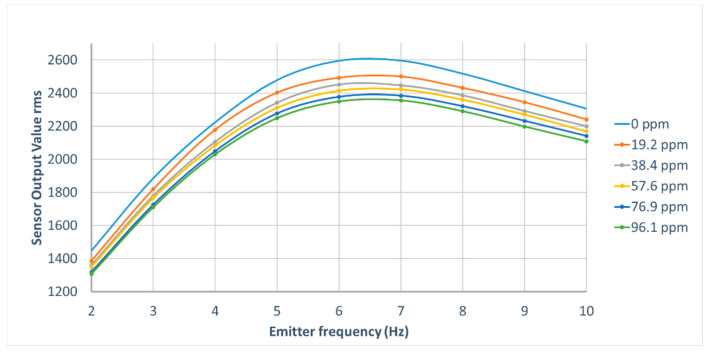
Variations of detector output values against the running frequency of the IR emitter.

The highest root means square (RMS) values of the detector output were found under 6 Hz (Figure 3). Standard deviations of the recorded detector RMS values fluctuated along each IR emitter operating frequency. The 6 Hz operating frequency showed higher energy incidence on the detector and was selected as the best operating level for the experiments.

### 3.2. Gas Detection Device Calibration and Model Validation

In the calibration process, the modified Bear-lambert law was applied to calculate the gas concentration from the detector output values. Accordingly, the equations with respect to the calculation of IR light absorbance followed by gas concentration is described as follows: Beer–Lambert law quantitatively explains the transmittance amount of monochromatic light through a gas [33]. Based on the gas concentration, the initial intensity of infrared on the active detector decreases according to an exponential relationship:

(1) $$I=I_{0}e^{-klx}$$

where:

*I* = intensity of the target gas;
*I*_0_ = intensity of zero gas;
*k* = absorption coefficient for the combination of a specific gas and filters;
*l* = equivalent optical path length;
*x* = concentration of gas.

Considering the changes of the output voltage of the active channel, the absorbance of incidence light by the gas is represented as

(2) $$ABS=\;\frac{\left(V_{o}-\;V\right)\;}{V_{o}}=\;\;\frac{\left(I_{o}-\;I\right)\;}{I_{o}}=\;1-\frac{I}{I_{o}}$$

where:

*ABS* = fractional absorbance;
$V_{o}$= output voltage of zero gas;
*V* = output voltage of the target gas.

By combining Equations (1) and (2), the fractional absorbance can be indicated as

(3) $$ABS=1-e^{-klx}$$

According to the Alphasence infrared sensor application notes, for practical application of NDIR, the Beer–Lambert law has been modified by adding the *SPAN* factor since all the incident light on the detector is not absorbed by the gas. The linearization power term “c”, added to compensate for the variations, is initiated due to optical path length and light scattering. The linearization coefficient “b” and SPAN values may depend on the concentration range of the measured gas [34,35,36]. Therefore, the formula for calculating absorbance is as follows:

(4) $$ABS=SPAN\left(1-e^{-bx^{c}}\right)$$

where:

*ABS* = absorbance;
*SPAN* = coefficient for the proportion of absorbance of the IR radiation;
*b* and *c* = linearization coefficients;
*x* = concentration of the gas.

Solving Equations (2) and (4), “*X*” concertation can be calculated by the following equation:

(5) $$X=\frac{T}{T_{LOW}}{\left[\frac{\ln\left(1-\frac{ABS}{SPAN}\right)}{-b}\right]}^{\left(\frac{1}{c}\right)}$$

where:

*T* = temperature of the gas in K at the sampling stage
*T_LOW_* = temperature of the gas at calibration

The temperature compensation (*T/TLOW*) fragment was added to the formula because of the concentration changes of the gas due to temperature variations (at the calibration stage, the equation assumes *T = TLOW*) [36].

The calibration process was performed by following the Pyreos AN0119 application notes [37]. We followed the zero-gas calibration method; N_2_ was used as the balance gas. Before initiating the measurements, the whole system was flushed by sending pure N_2_ gas for 5–10 min through the opened system, and it was enclosed after completion of proper ventilation by confirming the stability of sensor values. The pressure of the system was maintained at similar to the atmospheric pressure. During the calibration steps, a known volume of N_2_O gas was injected into an N_2_ filled external gas chamber, which was serially connected through a diaphragm air pump with the new gas detector and circulated through the system while recording the data. The detector output data were measured at N_2_O gas concentrations, ranging from 0–2000 ppm. Calculated values for fractional absorbance were determined by using Equation (2). Temporary values of absorbance were calculated from Equation (5) by assigning a value of 1 for all linearizing coefficients. The best-fitted values for linearizing coefficients were determined by running temporary absorbance values under the curve fitting program SLOVER tool of MS EXCEL software. The N_2_O gas concentration values at each level were calculated from Equation (5) by applying the observed linearizing coefficients. For validating the coefficient values, the regression graph was plotted between the calculated and injected pure gas concentrations.

#### Results of Device Calibration and Model Validation

**Figure 4:**
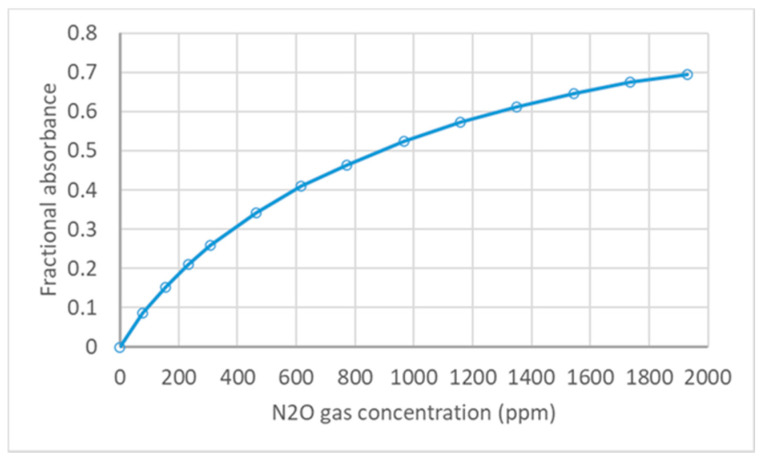
Nondispersive infrared (NDIR) fractional absorbance at each N_2_O gas concentration level.

**Figure 5:**
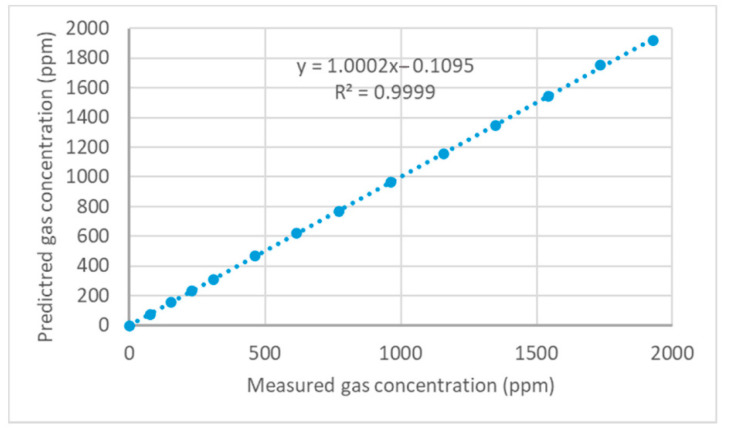
Regression graph of measured and predicted gas concentrations.

The graph in Figure 4 shows the variations in fractional absorbance of NDIR in the total measurable range of 1–2000 ppm. The curved graph of fractional absorbance was linearized, and gas concentrations were calculated by using Equation (5). Regression analysis on the measured and calculated gas concentration shows a 0.9999 coefficient of determination (R^2^) (Figure 5). The linearization coefficients of Equation (5) were determined as *SPAN*: 0.83477, *b*: 0.002619, *c*: 0.86352 for the 1–2000 ppm range of gas monitoring. For the second calibration curve on the selected soil gas monitoring range from 1 to 300 ppm, the linearization coefficients were *SPAN*: 0.672669, *b*: 0.001934, *c*: 0.968181. With these results, the low-cost design embedded with a non-reflective gas chamber isolated by optical windows, and the light beam, which was focused by a convex lens, has a great ability to measure the 1–2000 ppm range of gas concentrations for laboratory tests and 1–300 ppm monitoring levels for agricultural field evaluations.

### 3.3. Repeatability and Sensitivity Tests

Before monitoring the soil gas concentration in the laboratory, the developed calibration model was validated for reliability and consistency by following the analytical methods of the International Conference on Harmonization (ICH) [38,39] and published scientific validation methods [40]. The applied key statistical tools have sensitivity and repeatability, as described below.

Repeatability or reproducibility comprises the closeness of repeated measurement results from the same analyte obtained under the same method, laboratory, operator, and equipment. Repeatability tests are conducted in short time intervals; they reflect the best internal precision of the instrument, and normally the results are interpreted as the relative standard deviation, standard deviation, or coefficient of variation (CV). The relative standard deviation (% *RSD*) is calculated by dividing the standard deviation(s) of samples from sample set x average ($\bar{x}$) and multiplying by 100% as given in Equation (6):

(6) $$\begin{array}{ccc} \%\;RSD= & \frac{s}{\bar{X}} & \times 100\% \end{array}\;\;$$

Sensitivity refers to variations in the response of the analyzer to changes of analyte gas volume in the gas cell, determined by dividing the detector response (Δv) by the gas analyzer from the corresponding changes in analyte gas (Δg) at a given concentration of gas (g_0_) (Equation (7)):

(7) $$\begin{array}{cc} Sensitivity= & {\left(\frac{\Delta v}{\Delta g}\right)}_{g_{0}} \end{array}$$

The repeatability test was done by conducting 6 consecutive similar gas measurement events at the same laboratory. Each gas measurement event was performed by injecting 10 known levels (10–100 μL) of pure N_2_O gas into the gas chamber (volume 940 mL) that was serially connected with a circulation pump and gas analyzer (total system volume 1296 mL). The detector output data were recorded by circulating the gas through the gas detection device, and respective gas concentration levels were calculated using the calibration model with linearizing coefficients. Regression analysis was done for each measurement event to evaluate the accuracy of the calibration model. The repeatability was determined by calculating the% RSD (Equation (6)) from the recorded data. With the use of Equation (7), the instrument sensitivity was calculated from the obtained data with respect to the concentration levels starting from 3.38 ppm to 1929 ppm. The calculated sensitivity values were graphically extrapolated to indicate the variations along the applied gas concentration levels.

#### 3.3.1. Allan Variation for Signal Stability over the Operating Time

The Allan variance, known as two-sample variance, was initially developed by David W. Allan with the intention of determining the frequency stability of electronic components, such as clocks, oscillators, and amplifiers [41]. At present, it is commonly applied to illustrate the sensor noise and stability [28,42]. Accordingly, the Allan deviation plots display limits of detection as a function of integration time. The white noise made by the sensor and other parts of the electronic system, which determines the minimum detection limit, indicates the initial region of the plot [43]. The function of the Allan deviation (sigma-tau) is as follows:

(8) $$\sigma_{y}\left(\tau \right)=\;\frac{1}{\surd 2}\;\left({\bar{y}}_{n+1}-{\bar{y}}_{n}\right)$$

where:

$\sigma_{y}\left(\tau \right)$ = Allan deviation;
${\bar{y}}_{n}$ = *n*^th^ = Fractional signal average value belongs to the assigned time.

To determine the detection limit of a new device, the device was run at a constant gas concentration level at 7.7 ppm for a more than 10 min duration, and the data were recorded at a speed of 140 Hz. The Allan deviation plot was developed with the use of the R program, with the special package (avar: Allan variance) available on cran.r-project.org [44]. The minimum detection limit was determined from the Allan deviation plot.

#### 3.3.2. Results of Repeatability and Sensitivity of the Device Output

**Figure 6:**
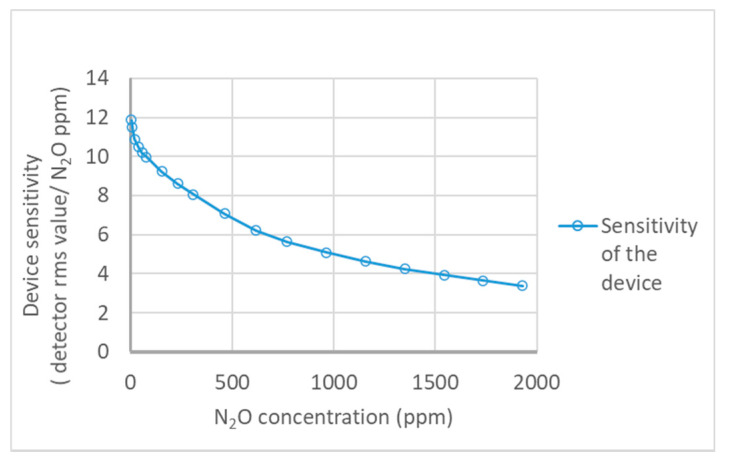
Sensitivity of the device at each N_2_O gas concentration levels.

**Figure 7:**
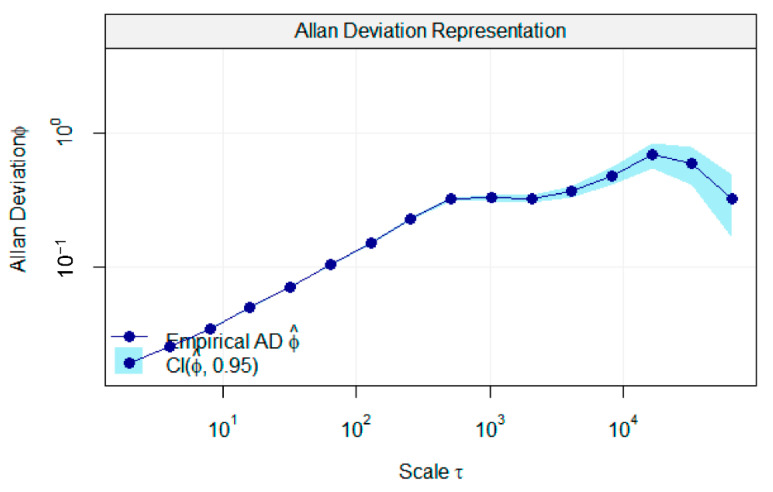
Allan deviations of instrument output at a 7.7 ppm N_2_O gas concentration.

Table 1 shows the results of repeatability tests on six testing events. The second calibration model was validated by the repeatability tests and indicates higher correlation coefficients (>0.9995) for each testing event by regression analysis. The maximum residual standard error, 0.627, was recorded. Therefore, the results show an embodied simple optical arrangement in the new NDIR gas analyzer operated under a higher accuracy level with repeated measurements. Figure 6 shows the variations of the device sensitivity along with applied gas concentrations. The maximum sensitivity was recorded as an 11.87 detector output RMS/ppm at the low concentration of N_2_O gas (3.87 ppm), and the minimum was a 3.38 detector output RMS/ppm at the high concentration (1929 ppm). Considering the instrument sensitivity data, it proves the ability to measure gas concentrations (<300 ppm) at a higher level of responsivity (>8.06 detector output RMS/ppm). The values of RSD% were less than 1.5%, and this indicates the best internal precision of the device under repeatability tests. Figure 7 shows the Allan deviations for continuous measurements recorded at a 7.7 ppm N_2_O gas concentration. Accordingly, the minimum detection limit of the instrument was observed as 1 ppm at the maximum deviation of the measured values.

### 3.4. Determination of the Impact on the Optical Path by the Humidity Level in the Gas

Since the spectrometer is to be used in field conditions, for long-term operations, the humidity level of air can be varied within the air circulation system. Although the moisture level of the air is not affected at the IR emitter or detector, which are isolated by an optical window, the impact of the humidity level of the gas chamber on the transmission of light energy should be considered. Therefore, the absorbance of light energy was measured with the changing of humidity levels (20–90%) in the gas–cell, while the instrument was running under applied pure N_2_O gas levels ranging from 19.2 to 76.8 ppm at each corresponding humidity level. To monitor the humidity level in the system, a humidity sensor (BME 280) installed mini chamber was serially connected with the other components of the gas monitoring system (monitoring device, air pump, gas chamber and two valves). For controlling the humidity levels, two separate sections (air-drying and humidifying) were parallelly connected to the gas monitoring system via four valves (two valves per section) and isolation of each section was performed through the valves. As the humidity absorber, the air-drying section was mainly based on silica gel, and humidifying section was with the water containers. During the events of drying and humidifying, while observing the humidity sensor data, the gas was sent through the dryer or the humidifier, depending on the event. After detecting the appropriate humidity level in the gas monitoring system, both drying and humidifying sections were isolated via the valves and required N_2_O gas volumes injected into the gas chamber.

#### Impact of the Humidity Level on Fractional Absorbance

**Figure 8:**
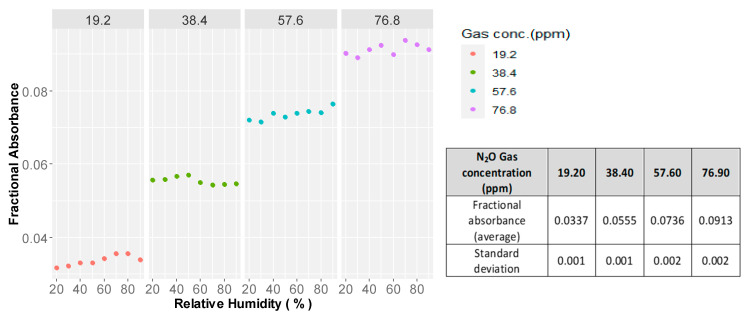
Fractional IR absorbance by N_2_O gas at different relative humidity levels.

The fractional absorbance of light is in the same cluster at each gas concentration level against the variations of relative humidity from 20–90% (Figure 8). The results of the ANOVA indicated that there was no significant impact (*p*-value 0.148 at a 95% confidence interval) of humidity level on light transmittance through the gas cell and the optical elements; a precision humidity control system is not necessary for gas monitoring by the developed instrument. Considering the long-term, closed-loop gas monitoring systems, gas diffused from the soil should be sent through a dehumidifying system to avoid condensation of water vapor on the optical elements and collection of water in the gas cell.

## 4. Practical Usability Evaluation

### 4.1. Test for the Gas-Accumulation Rate in the Silicone Diffusion Cell

For the soil-gas-concentration monitoring tests by the new device, for diffusing the generated N_2_O gas in the soil atmosphere into the gas monitoring system, a silicone tube was used as the diffusion cell. In this method, the time required to diffuse the gas from the soil to the silicone diffusion cell is important. The diffusion time should be shorter if possible to monitor a rapid change of gas concentration in the soil atmosphere, which could be an added advantage of the monitoring process. Therefore, the gas accumulation rate in the silicone diffusion cell connected with the new device and a reference device was tested separately. As an accurate reference, we used an FTIR (Perkin Elmer—Spectrum Two FT-IR spectrometer) plus a long path gas cell system (Infrared Analysis Inc., Anaheim, CA, USA, model 7.2-V; 7 m optical path length, volume 500 mL), which is often used to measure a low concentration of gases. Since the volumes of the new instrument (320.3 mL) and the reference instrument (500 mL) were different, the diffused N_2_O gas accumulation time in each gas monitoring system was tested. Accordingly, an 8000 mL glass container was used as the diffusion chamber, and a 590 mm length, 8 mm outer diameter and 6 mm inner diameter silicone tube was fixed on the lid as a closed-loop gas circulation pathway that opened to the gas measuring device. A volume controllable air pump and airflow meter were serially fixed into the gas circulation pathway. During the test, the diffusion chamber was filled with pure N_2_ and pure N_2_O (50 ppm), injected into the chamber through a rubber septum placed on the lid. The pump was run by adjusting the airflow rate to 1.66 × 10^−5^ m^3^ s^−1^, and the diffused N_2_O gas volume from the gas chamber to the silicone diffusion cell was measured by the new device and the reference device at each 30 min intervals. The recorded data were graphically interpreted, and the time for N_2_O gas accumulation was determined.

#### 4.1.1. N_2_O Gas-Accumulation Rate in the Diffusion Cell

In this experiment, compared to the initial N_2_O concentration (50 ppm) in the gas chamber, a lower concentration (33.32 ± 0.65 ppm) was detected in all silicone diffusion cells connected to both devices with 70.7% of maximum accumulation for the measured period. Jacinthe et al. [29] have described in their experiment the factors that affect the N_2_O accumulation rate in silicone tube as concentration gradient of target gas across the wall of the silicone tube and the diffusion coefficient of the silicone material at a given temperature. As such, in our experiment, the received accumulation values are fitted with the silicone material at 19.5 °C average room temperature. Therefore, although we did not test, the determination of the diffusion coefficients for the silicone material at respective operating temperature ranges is required to estimate the N_2_O flux in the soil region.

**Figure 9:**
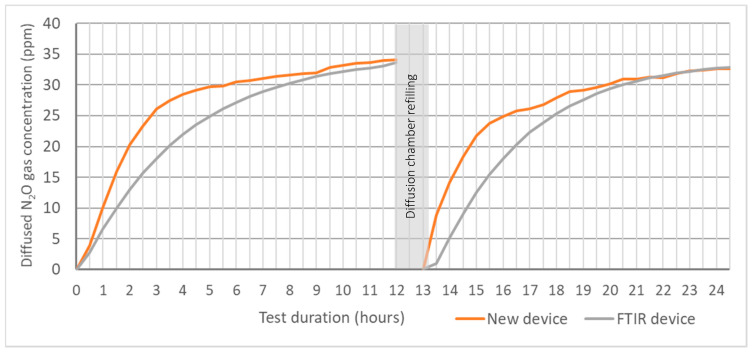
Diffusion rate of N_2_O gas into the sampling cell.

Mainly, two regions such as steep and moderate N_2_O accumulation rate are shown in the N_2_O accumulation curve (Figure 9). The steep accumulation region is highly important for soil gas monitoring purposes since it indicates a higher diffusion rate at a higher concentration gradient over the silicone diffusion cell wall. Considering the two gas-monitoring devices, the time consumption to pass the steep accumulation region by the new device is lower (3–4 h) than the FTIR device-connected system (7–8 h). Similar conditions are shown in the first and second testing events. This time variation occurred due to the higher volume, 500 mL, of the long path gas cell in the FTIR device compared to the volume of the new device of 320.3 mL. Therefore, the new device demonstrated an advantage in its low volume compared to the reference device due to its ability to give an early response at higher gas concentrations from the soil tests. 

### 4.2. Laboratory Test for Monitoring the N_2_O Gas Concentration in the Soil Atmosphere

**Figure 10:**
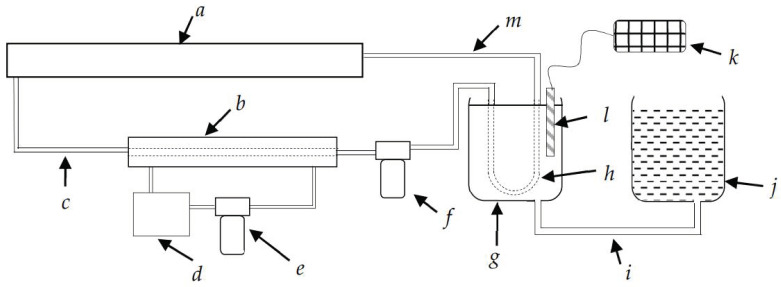
Experimental setup of the laboratory soil gas monitoring test. (*a*) Gas detecting device, (*b*) membrane dryer, (*c*) gas circulation tube, (*d*) silica gel container, (*e*) motor for dryer, (*f*) motor for gas circulation in the system, (*g*) soil container, (*h*) diffusion cell (silicone tube), (*i*) water supply tube, (*j*) water container, (*k*) data logger, (*l*) soil moisture probe, (*m*) gas circulation tube.

The new device should be tested for soil gas monitoring using a silicone diffusion cell. A laboratory test was conducted to monitor the gas concentration in the soil atmosphere. In this test, we checked whether the device could measure the temporal change of the N_2_O concentration in the soil gas. The soil sample, taken from the research field of the University of Ryukyus, was sieved by a 2 mm sieve and tested for the initial soil moisture level. As shown in Figure 10, the experimental setup mainly consisted of a serially interconnected air-drying section, soil container (*g*) and tubing (*c,m*), pumps (*e,f*), and the gas measuring device (*a*). The soil container (*g*) has a hole underneath and is connected to the water can (*j*) via a tube (*i*) to supply water from the downside during saturation events. The 1 kg of prepared soil was thoroughly mixed with 0.5 g of (NH_4_)_2_SO_4_ as an ammonium-based nitrogen source and placed into the soil container. A silicone tube diffusion cell (*h*) was buried in the soil to diffuse the N_2_O gas from the soil into the gas monitoring system, which was enclosed within the other components for gas circulation. The air pump (AS ONE-EAP-01) (*f*) circulates diffused air within the system. To avoid water accumulation in the system during long runs, the circulated air is first sent to the drying section. The drying section consists of a membrane-type dryer (Suncep SWG-A01-03) (*b*), which consists of two eccentric tubes—the middle one for soil gas circulation and the outward tube for dry gas circulation. In the drying section, the air pump (*e*) circulates dry gas within the silicone moisture absorber (*d*) and membrane dryer (*b*) in the opposite direction to the soil gas circulation. The gas monitoring device (*a*) is interconnected serially with all the components for circulating the soil–air through the tubes (*c*, *m*). The newly developed gas monitoring device, as a testing device, and the same FTIR spectrometer used in the previous experiment as a reference gas monitoring device, was connected to two separate soil gas monitoring systems, as indicated in Figure 10. A data-logger (*k*) connecting the moisture meter embedded within the EC and the thermometer (*l*) were placed in the soil container. The data of the two gas detecting devices and moisture meters were recorded at each 30 min intervals for 6 days. The water level of the container (*j*) was kept at the same level as the soil level (*g*) for one day for saturation, and the water container was kept below the level of the soil container to drain the excess water for two days; all three days were considered one cycle, and two cycles were completed. The recorded data were graphically extrapolated to demonstrate the variations of gas concentration levels in the soil atmosphere and the performances of each gas monitoring device.

#### Performance of the New Device in Laboratory Tests Monitoring the Soil Gas Concentration

**Figure 11:**
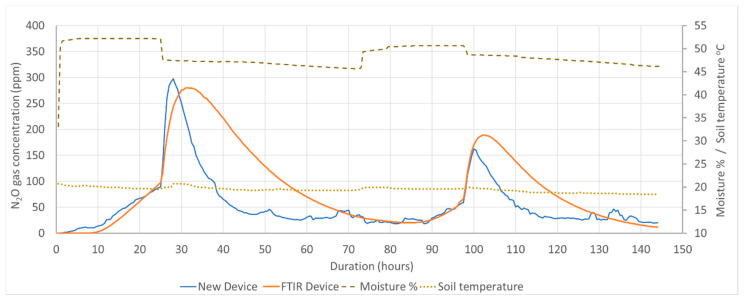
Recorded N_2_O gas concentrations by the new and reference devices.

Figure 11 shows the emitted N_2_O gas concentration levels at each 30 min intervals for two consecutive soil moisture saturation and drainage cycles. During the measurement period, the temperature variation in the soil environment has been indicated in Figure 11, and the average temperature was calculated as 19.5 ± 0.55 °C. Considering both cycles of soil moisture saturation, the highest gas concentration was recorded in the first cycle, as it was the immediate period of (NH_4_)_2_SO_4_ being added into the soil. During the initial stages of the first and second soil moisture saturation, both devices indicated similar values of gas concentrations as the emission rate of the soil was at a low-level, and adequate time had passed for diffusing and saturating the gas into the monitoring system. After starting the drainage, from the readings of the new device and FTIR device, the gas concentrations reached their peak in each cycle at 297.87 ppm, 280.12 ppm and 162.16 ppm, and 189.01 ppm in the first and second cycles, respectively. During the gas concentration measurements at accelerated soil gas production rate, considering the time taken to peak the gas concentration in each device, less time of 25–27 h was taken by the new device while 30–35 h consumed by FTIR device. The time consumption for equilibrating the gas level because of a higher ratio of the volume of the FTIR device to the diffusion cell is explained in Section 4.1.1. As such, the new device is more applicable for monitoring soil N_2_O gas concentrations in the soil atmosphere through the silicone diffusion cell gas sampling method.

## 5. Discussion

The silicone tube-based gas diffusion cell is more effective for continuous sampling, as the considerable emissions of soil N_2_O gas occur due to the application of nitrogen-based fertilizer, and monitoring activities should be carried out throughout the same time period (Figure 11). This is impractical using traditional gas chromatography followed by manual sampling [11]. The developed device has achieved continuous gas monitoring with the submerged silicone tube-based gas diffusion cell.

Considering the existing gas sampling techniques, the tested sampling system belongs to the passive gas sampling technique that develops concentration gradients of target gas within the soil environment. It allows continuous sampling with a higher temporal resolution than active sampling techniques, which are frequently used with low temporal resolution-required measurements [45]. In this method, with the diffusion coefficient of the material of the sampler, the development of a small-time lag and affecting hourly measurements are probable. By using the thin sampling material and reducing the volume of the measuring system, the time lag can be made shorter. Considering the applications in the real field, since the estimation of long-term emission is required, daily assessments on the emission are adequate with extended sampling intervals in which the influence of time lag can be neglected. In the measurements conducted in the sugarcane field, we have measured and confirmed the daily variations of N_2_O flux [46].

At present, highly sensitive laser-based spectrometers and FTIR devices are available for gas measurement; their power requirements, sensitivity in harsh environments, and initial and maintenance costs are barriers to the continuous monitoring of the soil atmosphere [47]. Compared to sophisticated devices, the developed device requires considerably less space (length: 750 mm, width: 80 mm, height: 80 mm) and weighs just 1.2 kg, giving it portability under field conditions. Since the power requirement of the new device is 9 V and 670 mA, the management of the power supply is easy. Table 2 shows the features and cost comparison on a developed device with commercially available, N_2_O gas measurable devices. Accordingly, the cost, size and power consumption of the developed device are significantly low and considered as beneficial features for replicated experiments that use multiple gas-monitoring devices in fields.

More specifically, the new device is easy to assemble and simple; it encourages researchers to monitor the soil N_2_O gas concentration by building their own, less expensive devices, and it facilitates easy maintenance, such as cleaning of the gas cell and optical components. Considering the instrument’s sensitivity and capacity, the minimum level it can measure is 1 ppm, which is adequate when measurements are conducted in the soil atmosphere since it contains higher gas concentration levels than the atmospheric level, which must be measured by highly sophisticated instruments. In this study, we applied 0.5 g of ammonium sulfate to 1 kg of soil and received a maximum level of 297.87 ppm of N_2_O gas concentration. The maximum measuring range of 2000 ppm of the new device allows to carry out a higher rate of fertilizer application in trials at the laboratory level.

## 6. Conclusions

We have developed a simple, easy-to-assemble, low-cost NDIR device for detecting soil N_2_O concentrations through a submerged diffusion cell. We used commercially available optical and optomechanical components, a MEMS-based IR source, a detector, and a microcontroller unit for the new device. The optical path length of 59 cm and 320.3 mL of the volume contained in the gas cell covered with two optical windows and one convex lens achieved a 1–2000 ppm measurable range with a maximum sensitivity of an 11.87 detector output RMS/ppm at the low concentration (3.87 ppm) of N_2_O gas, and the minimum was a 3.38 detector output RMS/ppm at the high concentration (1929 ppm). Instrument calibration and model validation, followed by a repeatability test, found 0.9995 of R^2^ with less than 1.5% of the RSD%. The results of the laboratory soil experiment setup revealed that the developed device has a great ability to measure the N_2_O gas presence in the soil atmosphere through a silicone tube-based submerged diffusion cell.

## Figures and Tables

**Table 1:** Results of the repeatability test of the new device.

	Repeatability					
Testing Event	D1	D2	D3	D4	D5	D6
Tested gas concentration range (ppm)	1–100					
Number of samples (n) per each concentration	3898	3898	3898	3898	3898	3898
Standard deviation (Sd) maximum	0.810	0.798	0.903	0.940	0.940	0.935
Standard deviation (Sd) mean	0.344	0.312	0.382	0.356	0.320	0.310
Residual standard error	0.5504	0.4975	0.627	0.567	0.5395	0.5133
RSD% (at 7.7 ppm N_2_O)	0.79	1.06	0.72	0.97	1.44	1.32
Correlation coefficient (r)	0.9996	0.9997	0.9995	0.9996	0.9997	0.9997

**Table 2:** Comparison in cost and other specifications of the developed device with commercially available gas analyzers.

	Gas Monitoring Device	Applied Technology	Type of Gas	Measuring Range	Dimensions (Length × Width × Height in mm), Weight (kg)	Power Requirement	Approximate Initial Cost (US Dollar)
	Developed device	NDIR	N_2_O	1–2000 ppm	750 × 80 × 80, 1.2	9 V, 670 mA DC	2780
[48]	Innova 1314i	* PAS	Multi-gas	From sub-ppm level to above	483 × 375 × 175, 14	100–240 V AC	40,000
[49]	Gasmet -DX4040 Portable gas Analyzer	FTIR	Multi-gas (up to 25 gasses)	From sub-ppm level to above	360 × 200 × 150, 13.8	230 V AC/2.5 h Battery power	65,000
[50]	PerkinElmer—Spectrum Two FT-IR (plus a long path gas cell)	FTIR	Multi-gas	From sub-ppm level to above	450 × 300 × 210, 13	100–240 V AC	37,000

* photoacoustic spectroscopy.

## Data Availability

Data sharing is not applicable to this article.
